# Supplementary material for: Smooth muscle FGF/TGFβ cross talk regulates atherosclerosis progression
Source: EMBO Mol Med. 2016 May 13;8(7):712–28. doi: 10.15252/emmm.201506181 (PMC4931287; doi:10.15252/emmm.201506181)
Supplement: Supplementary file 1 — Appendix [file EMMM-8-712-s001.pdf]

Smooth muscle FGF/TGFβ cross-talk regulates atherosclerosis progression

P-Y. Chen et al., Appendix

Table of contents:

Appendix Figure S1 and figure legend

Appendix Figure S2 and figure legend

Appendix Table S1

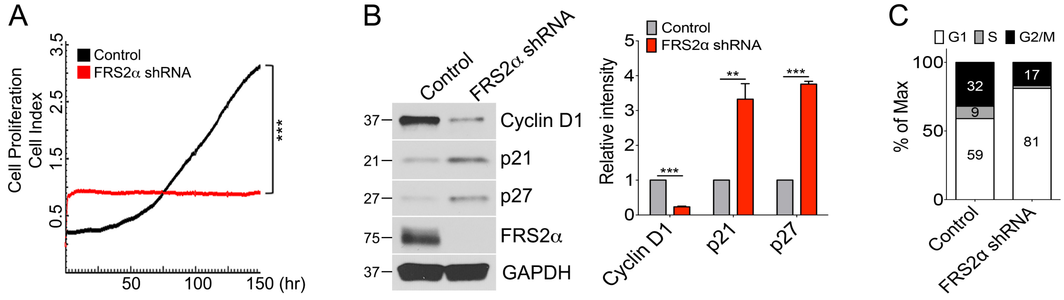

Appendix Figure S1. FRS2α knockdown inhibits proliferation of human aortic smooth muscle cells (HASMCs).

(A) Control and FRS2α knockdown HASMCs were cultured in the growth medium (M231 + SMGS). Cell proliferation was analyzed using real-time cell analysis (xCELLigence). Cell proliferation curves are representative of three independent experiments (***p<0.001 compared to control; unpaired two-tailed Student's t test).

(B) Left: Control and FRS2α knockdown HASMCs were cultured in the growth medium (M231 + SMGS). Immunoblot analysis of cell cycle regulators Cyclin D1, p21, and p27 in control and FRS2α knockdown HASMCs. Blots are representative of four independent experiments. Right: Band intensities of Cyclin D1, p21, and p27 were normalized to GAPDH and expressed as a fraction of a control value. Results are expressed as means ± SD (**p<0.01; ***p<0.001 compared to control; unpaired two-tailed Student's t test).

(C) Control and FRS2α knockdown HASMCs were cultured in the growth medium (M231 + SMGS). Flow cytometry analysis with propidium iodide (PI) staining was used to evaluate the percentage of cellular DNA content in control and FRS2α knockdown HASMCs. Histogram of cell cycle distribution results are representative of three independent experiments.

Source data are available online for this figure.

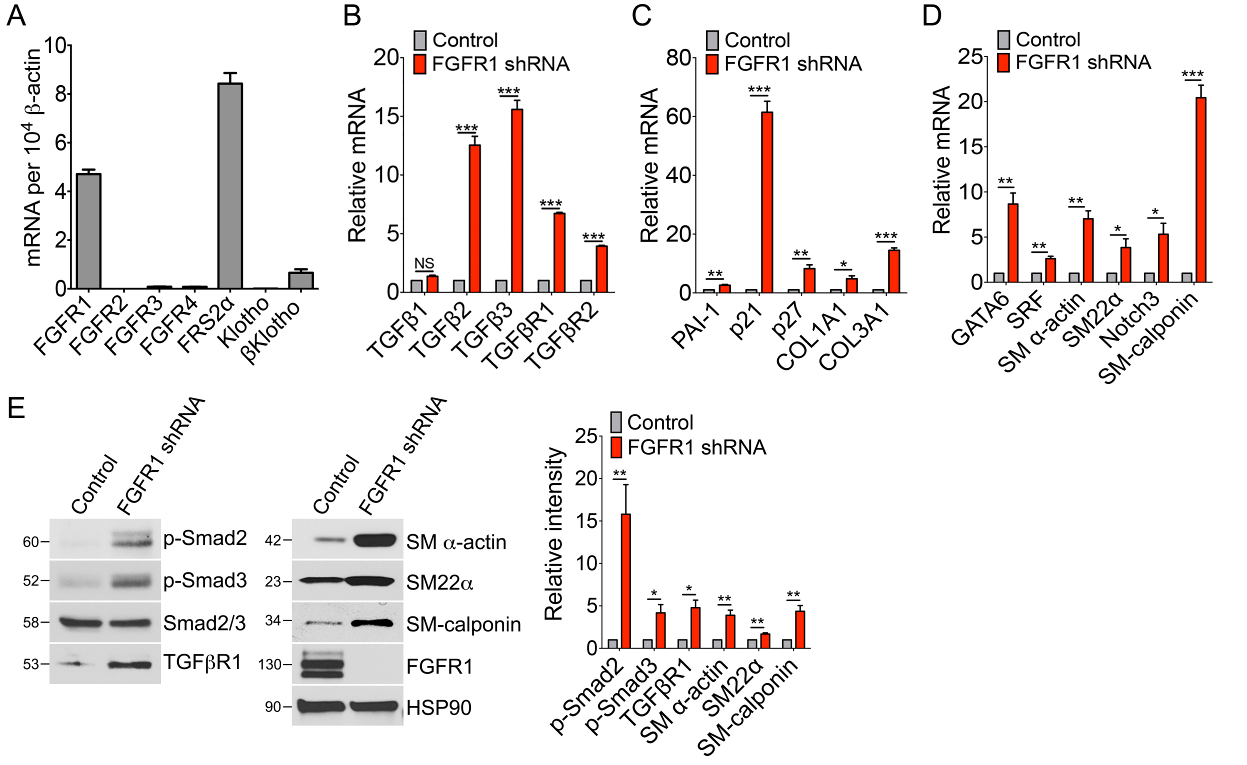

Appendix Figure S2. FGFR1 knockdown activates TGFβ signaling and induces smooth muscle marker gene expression in primary human aortic smooth muscle cells (HASMCs).

(A) qRT-PCR analysis of *FGFRs*, *FRS2α*, and *Klotho* family gene expression in primary human aortic smooth muscle cells (HASMCs). Data are presented as mean ± SD. β-actin was used for sample loading normalization. Histogram of qRT-PCR results are representative of four independent experiments.

(B-C) qRT-PCR analysis of TGFβ ligands, TGFβ receptors, and downstream target genes in control and FGFR1 knockdown HASMCs. (*p<0.05; **p<0.01; ***p<0.001 compared to control; unpaired two-tailed Student's t test). β-actin was used for sample loading normalization. Histogram of qRT-PCR results are representative of three independent experiments.

(D) qRT-PCR analysis of smooth muscle cell transcription factors and smooth muscle marker gene expression in control and FGFR1 knockdown HASMCs. (*p<0.05; **p<0.01; ***p<0.001

compared to control; unpaired two-tailed Student's t test. N=3). β-actin was used for sample loading normalization.

(E) Left: Immunoblot analysis of TGFβ signaling, TGFβ downstream targets, and smooth muscle markers in control and FGFR1 knockdown HASMCs. Blots are representative of four independent experiments. Right: Band intensities of p-Smad2, p-Smad3, TGFβR1, and SM α-actin, SM22α, and SM-calponin were normalized to Smad2/3 or HSP90 and expressed as a fraction of a control value. Results are expressed as means ± SD (*$p<0.05$; **$p<0.01$ compared to control; unpaired two-tailed Student's t test).

Source data are available online for this figure.

Appendix Table S1

Figure	Panel	Comparison	Target name	p-values
Figure 1	A	Control vs. FRS2α shRNA	TGFβ1	0.21068953
			TGFβ2	0.008319712
			TGFβ3	0.01072382
			TGFβR1	0.00025979
			TGFβR2	0.015268231
	B	Control vs. FRS2α shRNA	CTGF	0.000759988
			Elastin	0.01246853
			PAI-1	0.028557386
			p21	0.009203011
			p27	0.006670925
			COL1A1	0.004211705
			COL3A1	0.002138535
	C	Control vs. FRS2α shRNA	p-Smad2	8.88493E-05
			p-Smad3	0.000930205
			TGFβR1	0.000721485
			TGFβR2	0.020160803
Figure 2	A	Control vs. FRS2α shRNA	SM α-actin	0.000101378
			SM22α	0.009544707
			SM-calponin	0.000470604
	B	Control vs. FRS2α shRNA	GATA6	0.003158055
			MyoCD	0.019332723
			SRF	0.000256963
			MKL1	0.042464947
			MKL2	0.00583175
	C	Control vs. FRS2α shRNA		0.019792497
Figure 3	A	Control vs. FRS2α shRNA	*let-7*a	8.61615E-06
			*let-7*b	0.001884575
			*let-7*c	3.00833E-05
			*let-7*d	0.000531204
			*let-7*e	0.00029115
			*let-7*f	0.000654963
			*let-7*g	0.002539428
			*let-7*i	2.50215E-05
			miR-98	2.28407E-05
Figure 4	F	No/mild vs. Moderate	p-FGFR1	< 0.0001
		No/mild vs. Severe	p-FGFR1	< 0.0001
	H	No/mild vs. Moderate	FGFR1	0.4531
		No/mild vs. Severe	FGFR1	0.5424
Figure 5	B	No/mild vs. Moderate	TGFβ	< 0.0001
		No/mild vs. Severe	TGFβ	< 0.0001
	D	No/mild vs. Moderate	p-Smad2	< 0.0001
		No/mild vs. Severe	p-Smad2	< 0.0001
	F	No/mild vs. Moderate	p-Smad3	< 0.0001
		No/mild vs. Severe	p-Smad3	< 0.0001
Figure 6	G	ND vs. HFD	p-FGFR1	1.64955E-07
	H	ND vs. HFD	FGFR1	0.746861115
	I	ND vs. HFD	p-Smad2	4.21692E-08
	J	ND vs. HFD	p-Smad3	5.25794E-07
Figure 7	B	$Apoe^{-/-}$ vs. $Frs2\alpha^{SMCKO}/Apoe^{-/-}$		1.23382E-05

	F	$Apoe^{-/-}$ vs. $Frs2\alpha^{SMCKO}/Apoe^{-/-}$		1.09095E-05
	G	$Apoe^{-/-}$ vs. $Frs2\alpha^{SMCKO}/Apoe^{-/-}$	Fibrous cap	0.035372796
		$Apoe^{-/-}$ vs. $Frs2\alpha^{SMCKO}/Apoe^{-/-}$	Necrotic core	0.002780126
	H	$Apoe^{-/-}$ vs. $Frs2\alpha^{SMCKO}/Apoe^{-/-}$	$Ki67^{+}$ in plaque	0.000273109
		$Apoe^{-/-}$ vs. $Frs2\alpha^{SMCKO}/Apoe^{-/-}$	$Ki67^{+}$ in media	0.017889301
Figure EV1	A	Control DMSO vs.<br>Control SB431542	SB431542	0.00599426
		FRS2α shRNA vs.<br>FRS2α shRNA SB431542	SB431542	1.8648E-05
		Control vs.<br>TGFβR2 shRNA	TGFβR2 shRNA	0.004766565
		FRS2α shRNA vs.<br>FRS2α shRNA TGFβR2 shRNA	TGFβR2 shRNA	6.48404E-05
		Control vs.<br>Smad2 shRNA	Smad2 shRNA	0.000479918
		FRS2α shRNA vs.<br>FRS2α shRNA Smad2 shRNA	Smad2 shRNA	5.38782E-05
	C	Control DMSO vs.<br>Control SB431542	SB431542	0.009199739
		FRS2α shRNA vs.<br>FRS2α shRNA SB431542	SB431542	0.039178636
		Control vs.<br>TGFβR2 shRNA	TGFβR2 shRNA	0.002649561
		FRS2α shRNA vs.<br>FRS2α shRNA TGFβR2 shRNA	TGFβR2 shRNA	4.44002E-06
		Control vs.<br>Smad2 shRNA	Smad2 shRNA	0.000455524
		FRS2α shRNA vs.<br>FRS2α shRNA Smad2 shRNA	Smad2 shRNA	0.004117991
Figure EV3	A	Control vs. $Frs2\alpha^{SMCKO}$	*Frs2α*	3.66067E-05
	F	Control vs. $Frs2\alpha^{SMCKO}$	p-Smad2	0.069308226
	G	Control vs. $Frs2\alpha^{SMCKO}$	CD31	0.80468638
	H	Control vs. $Frs2\alpha^{SMCKO}$	CD31	0.074908489
Figure EV4	A	$Apoe^{-/-}$ vs. $Frs2\alpha^{SMCKO}/Apoe^{-/-}$	Body weight<br>before diet	0.672073639
		$Apoe^{-/-}$ vs. $Frs2\alpha^{SMCKO}/Apoe^{-/-}$	Body weight<br>after diet	0.618178759
	B	$Apoe^{-/-}$ vs. $Frs2\alpha^{SMCKO}/Apoe^{-/-}$	Total cholesterol<br>before diet	0.779589758
		$Apoe^{-/-}$ vs. $Frs2\alpha^{SMCKO}/Apoe^{-/-}$	Total cholesterol<br>after diet	0.436595645
		$Apoe^{-/-}$ vs. $Frs2\alpha^{SMCKO}/Apoe^{-/-}$	Triglycerides<br>before diet	0.509710362
		$Apoe^{-/-}$ vs. $Frs2\alpha^{SMCKO}/Apoe^{-/-}$	Triglycerides<br>after diet	0.765199153
		$Apoe^{-/-}$ vs. $Frs2\alpha^{SMCKO}/Apoe^{-/-}$	HDL-C<br>before diet	0.702547808
		$Apoe^{-/-}$ vs. $Frs2\alpha^{SMCKO}/Apoe^{-/-}$	HDL-C<br>after diet	0.541724025
	C	$Apoe^{-/-}$ vs. $Frs2\alpha^{SMCKO}/Apoe^{-/-}$	Aorta diameter	0.800887669
	D	$Apoe^{-/-}$ vs. $Frs2\alpha^{SMCKO}/Apoe^{-/-}$	Cardiac output	0.648203508
		$Apoe^{-/-}$ vs. $Frs2\alpha^{SMCKO}/Apoe^{-/-}$	Ejection fraction	0.729668945
		$Apoe^{-/-}$ vs. $Frs2\alpha^{SMCKO}/Apoe^{-/-}$	Fractional shortening	0.677064475

Figure EV5	B	Apoe$^{-/-}$ vs. *Frs2α*^{SMCKO}/Apoe$^{-/-}$		0.001568785
	C	Apoe$^{-/-}$ vs. *Frs2α*^{SMCKO}/Apoe$^{-/-}$	SM α-actin	0.010162341
	D	Apoe$^{-/-}$ vs. *Frs2α*^{SMCKO}/Apoe$^{-/-}$	Collagen 1	0.003401397
Appendix Figure S1	A	Control vs. FRS2α shRNA		2.30504E-08
	B	Control vs. FRS2α shRNA	Cyclin D1	2.85811E-06
		Control vs. FRS2α shRNA	p21	0.00634715
		Control vs. FRS2α shRNA	p27	4.63817E-06
Appendix Figure S2	B	Control vs. FGFR1 shRNA	TGFβ1	0.057202263
			TGFβ2	0.000411123
			TGFβ3	0.000201609
			TGFβR1	2.35635E-05
			TGFβR2	0.000562039
	C	Control vs. FGFR1 shRNA	PAI-1	0.004098649
			p21	8.52174E-05
			p27	0.004984374
			COL1A1	0.019002459
			COL3A1	9.19268E-05
	D	Control vs. FGFR1 shRNA	GATA6	0.00339833
			SRF	0.003614581
			SM α-actin	0.002391361
			SM22α	0.042986096
			Notch3	0.023726067
			SM-calponin	0.000150021
	E	Control vs. FGFR1 shRNA	p-Smad2	0.005352937
			p-Smad3	0.030159117
			TGFβR1	0.018717934
			SM α-actin	0.00132619
			SM22α	0.002987686
			SM-calponin	0.003242258
