# Supplementary material for: Smooth muscle FGF/TGFβ cross talk regulates atherosclerosis progression
Source: EMBO Mol Med. 2016 May 13;8(7):712–28. doi: 10.15252/emmm.201506181 (PMC4931287; doi:10.15252/emmm.201506181)
Supplement: Supplementary file 2 — Expanded View Figures PDF [file EMMM-8-712-s002.pdf]

Expanded View Figures

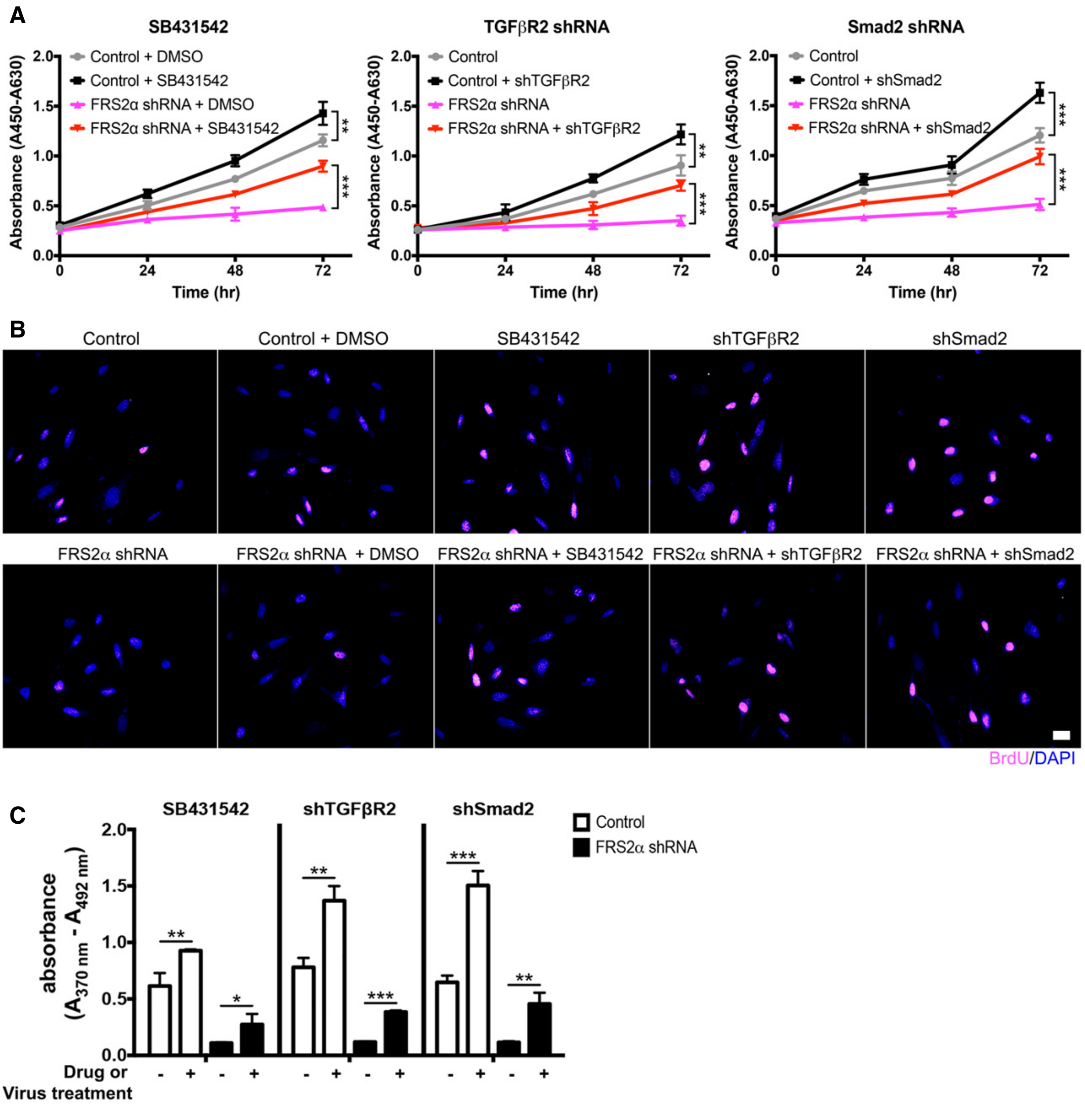

Figure EV1. Inhibition of TGFβ signaling is able to reverse FRS2α-knockdown-induced growth arrest phenotype in primary human aortic smooth muscle cells (HASMCs).

A Human aortic smooth muscle cells treated with FRS2α shRNA, TGFβR2 shRNA, Smad2 shRNA, or SB431542 (10 μM) were subjected to cell proliferation assay. Cell proliferation was measured by WST-1 assay. Data are means ± SD from triplicates in each group from three independent experiments (**$P < 0.01$, ***$P < 0.001$; unpaired two-tailed Student's *t*-test).

B Immunofluorescence staining of BrdU (red) in control, FRS2α shRNA-, TGFβR2 shRNA-, Smad2 shRNA-, and SB431542 (10 μM)-treated HASMCs. Nuclei were counterstained with DAPI (blue). Scale bar: 16 μm. Images are representative of three independent experiments.

C Cell proliferation was quantified by using Cell Proliferation ELISA BrdU Assay (Sigma). Cells were labeled with BrdU (10 μM) for 2 h. BrdU incorporation was determined by ELISA and the OD_{450}-OD_{690} was determined. Data are mean ± SD from triplicates in each group from three independent experiments (*$P < 0.05$, **$P < 0.01$, ***$P < 0.001$; unpaired two-tailed Student's *t*-test).

Data information: A full table of *P*-values for this figure is shown in Appendix Table S1.

 © 2016 The Authors

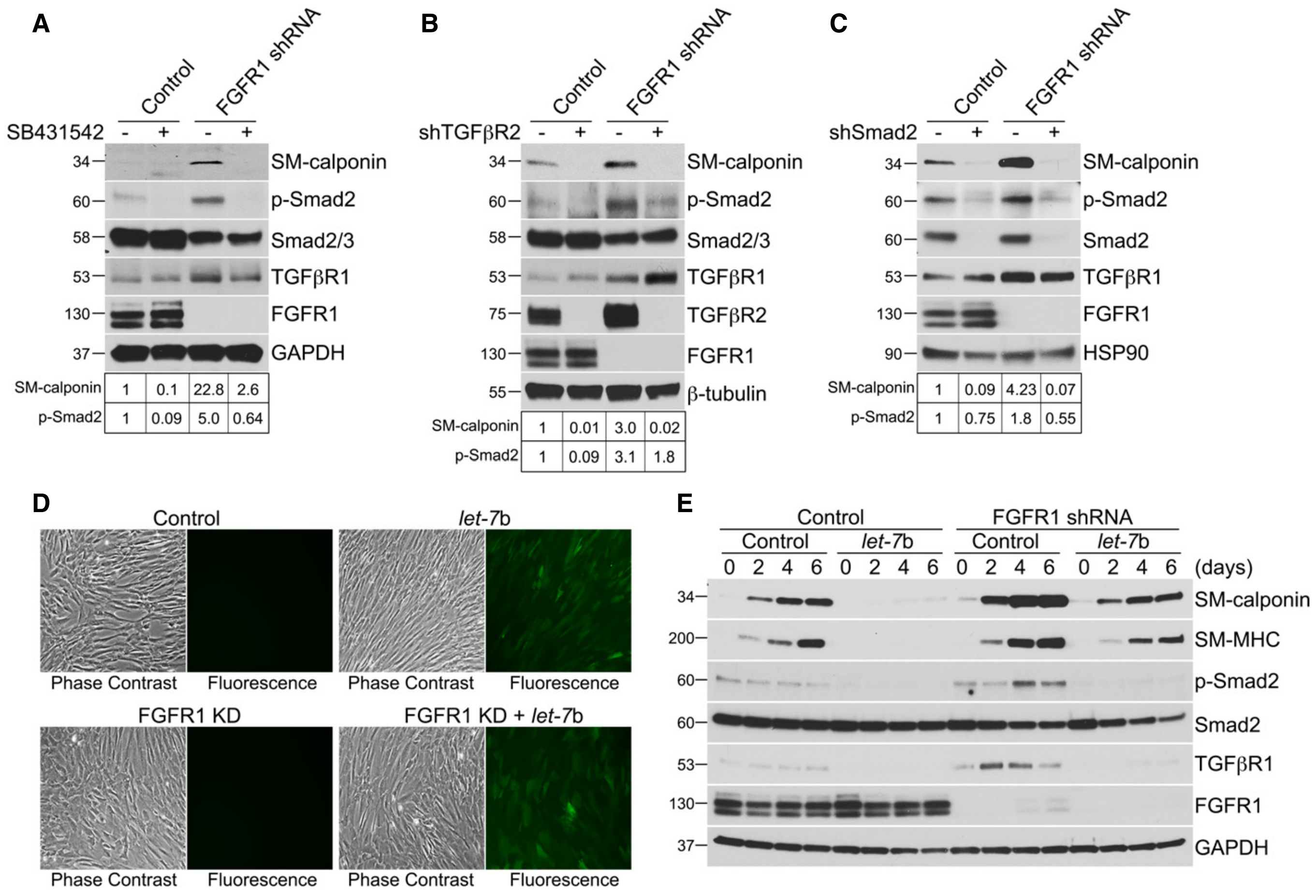

Figure EV2. FGFR1 knockdown increases smooth muscle marker gene expression via the *let-7*-TGFβ pathway in primary human aortic smooth muscle cells (HASMCs).

A–C Upper panels: Immunoblots of SM-calponin, phosphorylated Smad2 (p-Smad2), and TGFβR1 expression in control and FGFR1-knockdown HASMCs treated with SB431542 (10 μM), TGFβR2, or Smad2 shRNA lentiviruses. Blots are representative of three independent experiments. Bottom panels: Band intensities of SM-calponin and p-Smad2 were normalized to GAPDH, β-tubulin, HSP90, or Smad2/3 and expressed as a fraction of a control value.

D Images of phase-contrast and GFP fluorescence signals showed the expression of *let-7*b at day 0. Images are representative of three independent experiments.

E Control and FGFR1-knockdown HASMCs were cultured in the growth medium (M231+ SMGS) at day 0 and then switched from growth conditions to differentiation medium (M231+ SMDS) for 6 days with or without *let-7*b lentiviruses. Immunoblots of smooth muscle markers, phosphorylated Smad2 (p-Smad2), and TGFβR1 expression in control and FGFR1-knockdown HASMCs with or without *let-7*b lentiviruses. Blots are representative of three independent experiments.

Source data are available online for this figure.

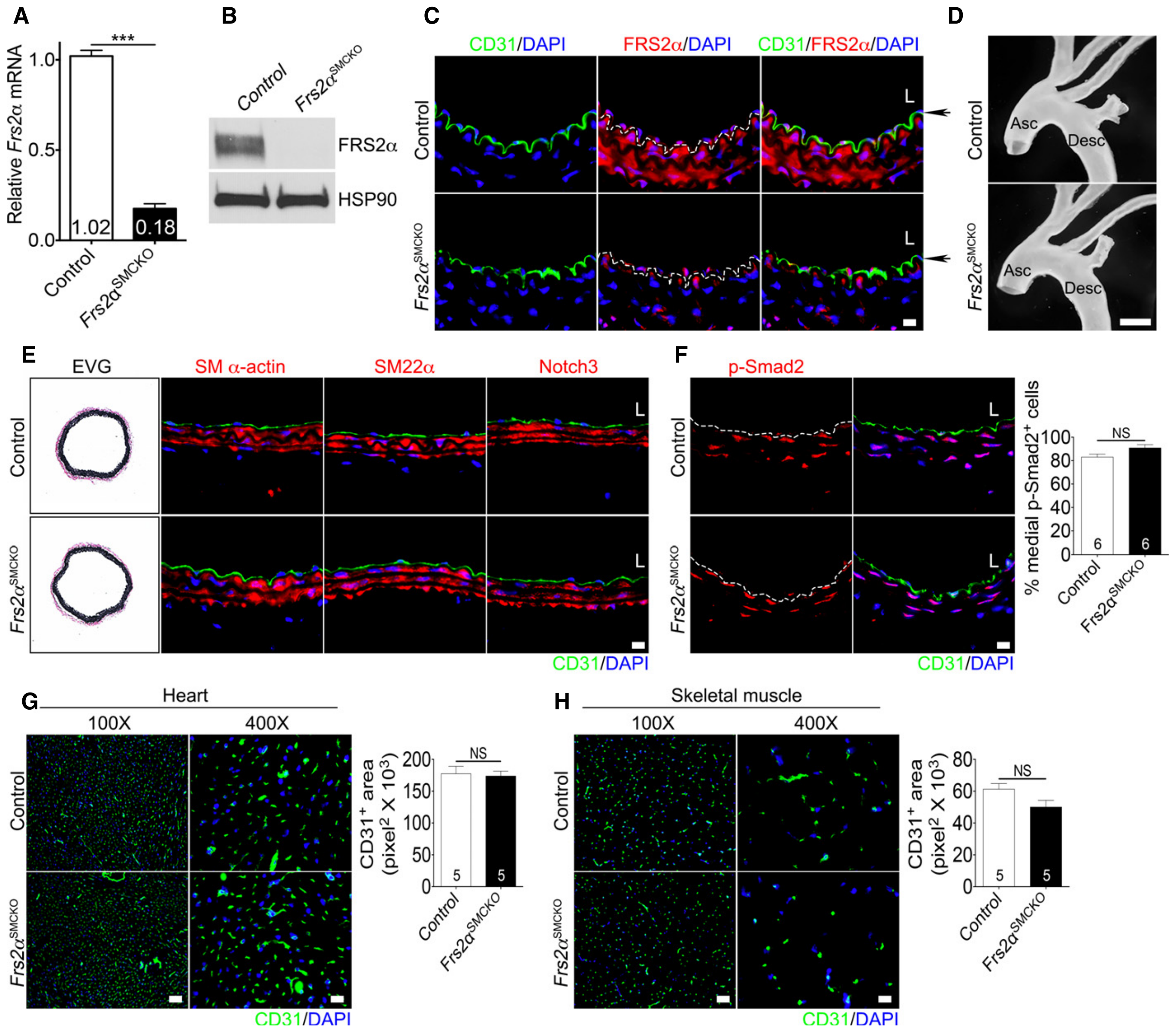

Figure EV3. *Frs2α^SMCKO* mice display normal vascular morphology and vascular density.

A qRT–PCR analysis of *Frs2α* expression in mouse aorta. β-actin was used for sample loading normalization. $n = 3$ control and 3 *Frs2α*^SMCKO mice were analyzed.

B Immunoblot analysis of FRS2α expression in mouse aorta. In each group, aorta was pooled from 4 mice/group.

C Representative images of FRS2α immunofluorescence staining of control and *Frs2α*^SMCKO aorta. Endothelial cells are visualized by CD31 (green). Black arrows indicate endothelial cells. L: lumen. Nuclei were stained with DAPI (blue). Images are representative of 3 mice/group. Scale bar: 10 μm.

D Gross appearance of aorta in 8-week-old control and *Frs2α*^SMCKO mice. Asc: Ascending; Desc: Descending. Images are representative of 3 mice/group. Scale bar: 5 mm.

E 5-μm cross sections of control and *Frs2α*^SMCKO mouse brachiocephalic artery were stained with EVG (elastic Van Gieson), anti-SM α-actin, anti-SM22α, and anti-Notch3 antibodies. Nuclei were counterstained with DAPI (blue). L: lumen. Scale bar: 10 μm. Images are representative of 3 mice/group.

F Left: Histological analysis of control and *Frs2α*^SMCKO mouse brachiocephalic artery with anti-CD31 (green) and anti-p-Smad2 (red) antibodies. Nuclei were counterstained with DAPI (blue). L: lumen. Scale bar: 10 μm. Right: Percentage of p-Smad2$^+$ cells in the media. Images are representative of 6 mice/group.

G, H Left: Representative images of vascular structure in heart and skeletal muscle in control and *Frs2α*^SMCKO mice. Scale bar: 62 μm for 100× and 16 μm for 400×. Right: Vascular density was quantified. Images are representative of 5 mice/group.

Data information: All data represent the mean ± SD (NS: not significant compared to control; ***$P < 0.001$ compared to control; unpaired two-tailed Student's *t*-test). Source data are available online for this figure.

 © 2016 The Authors

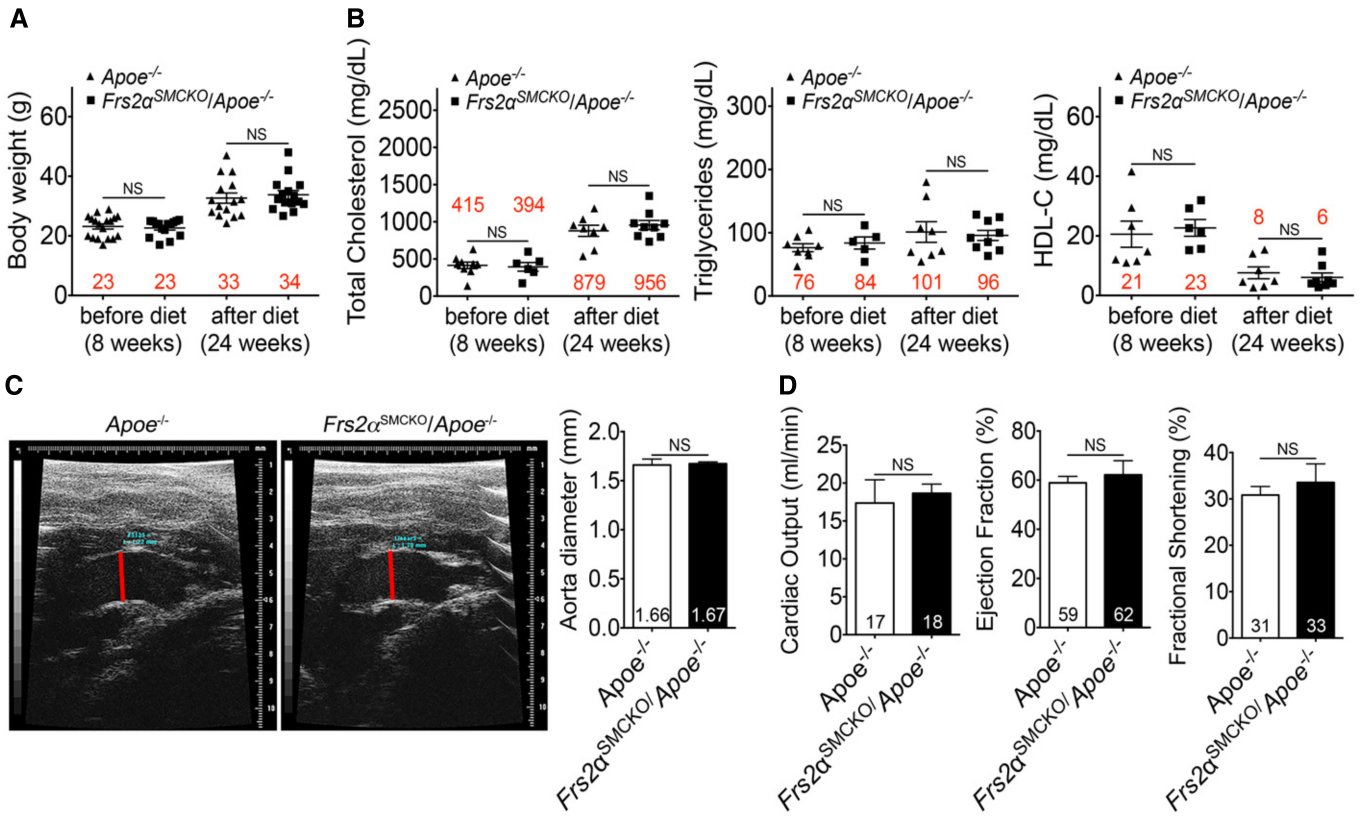

Figure EV4. ***Frs2α*^{SMCKO}/Apoe$^{-/-}$ mice have normal body weight, lipid profiles, and heart function.**

A, B Body weight, total cholesterol, triglycerides, and HDL-C analysis of *Apoe*$^{-/-}$ and *Frs2α*^{SMCKO}/*Apoe*$^{-/-}$ mice before and after 16 weeks on a high-cholesterol diet.

C Representative ultrasound images and ascending aorta diameters (red lines) of *Apoe*$^{-/-}$ and *Frs2α*^{SMCKO}/*Apoe*$^{-/-}$ mice. n = 3 *Apoe*$^{-/-}$ and 3 *Frs2α*^{SMCKO}/*Apoe*$^{-/-}$ mice were analyzed.

D Echocardiographic analysis in *Apoe*$^{-/-}$ and *Frs2α*^{SMCKO}/*Apoe*$^{-/-}$ mice showed no effect on cardiac output, ejection fraction, and fractional shortening. n = 3 *Apoe*$^{-/-}$ and 3 *Frs2α*^{SMCKO}/*Apoe*$^{-/-}$ mice were analyzed.

Data information: All data represent the mean ± SD (NS: not significant compared to *Apoe*$^{-/-}$; unpaired two-tailed Student's t-test). A full table of P-values for this figure is shown in Appendix Table S1.

© 2016 The Authors

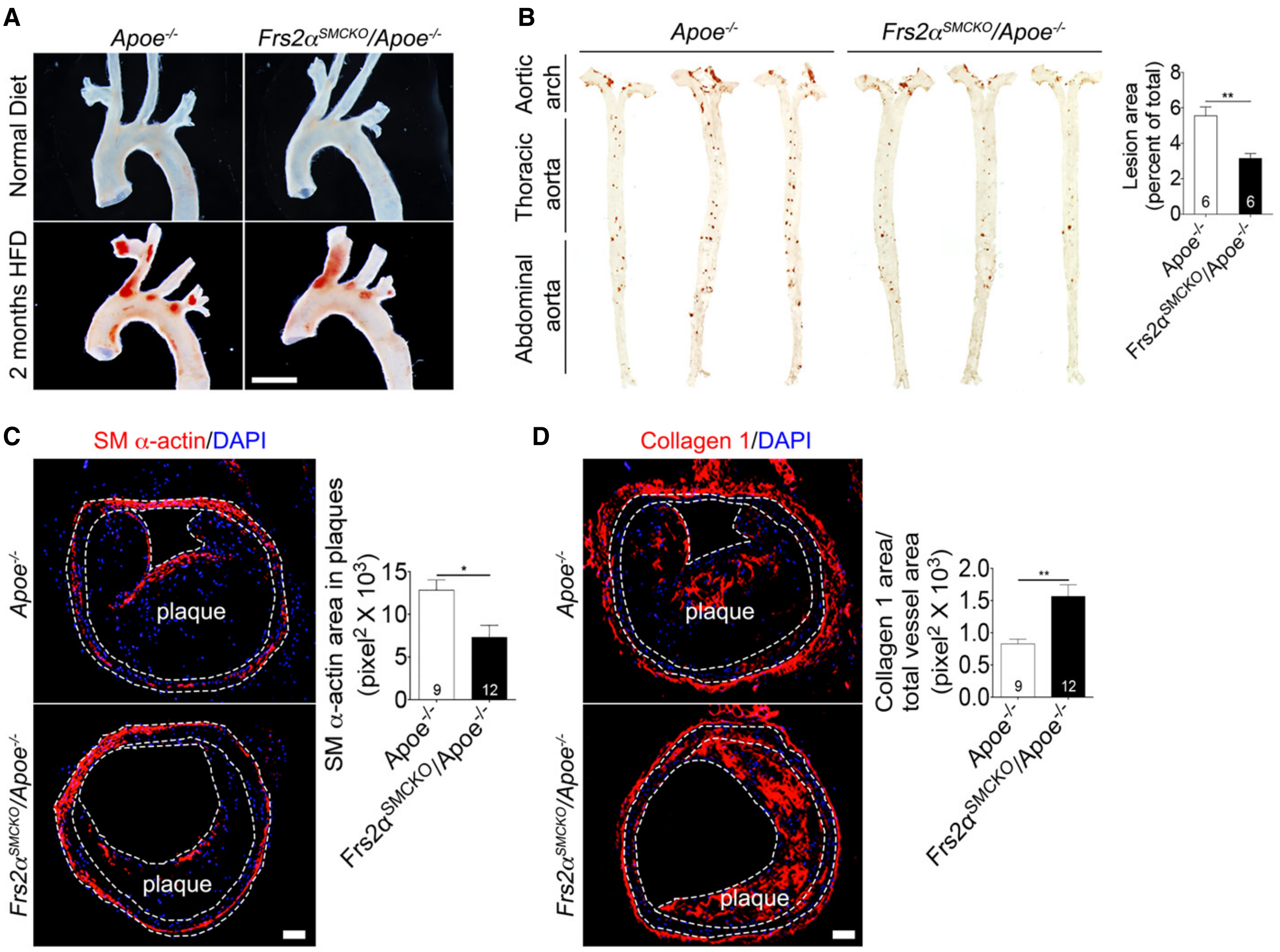

Figure EV5. Smooth muscle cell *Frs2α* knockout inhibits atherosclerosis plaque development.

- A Representative photomicrographs of Oil Red O-stained atherosclerotic lesions in the aortic arch of *Apoe*$^{-/-}$ or *Frs2*^{SMCKO}/*Apoe*$^{-/-}$ mice after 2 months of high-fat diet or normal diet. Images are representative of 3 mice/group. Scale bar: 5 mm.
- B (Left) Microphotographs of aortas (en face) from *Apoe*$^{-/-}$ and *Frs2*^{SMCKO}/*Apoe*$^{-/-}$ mice after 2 months of high-fat diet after staining with Oil Red O. (Right) Lesion area quantification. n = 6 mice per group.
- C Quantification of SM α-actin area in the plaque from *Apoe*$^{-/-}$ and *Frs2*^{SMCKO}/*Apoe*$^{-/-}$ mice after 4 months of high-fat diet. *Apoe*$^{-/-}$ mice N = 9, *Frs2*^{SMCKO}/*Apoe*$^{-/-}$ mice N = 12. Nuclei were counterstained with DAPI (blue). Scale bar: 62 μm.
- D Measurement of collagen 1 area from *Apoe*$^{-/-}$ and *Frs2*^{SMCKO}/*Apoe*$^{-/-}$ mice after 4 months of high-fat diet (**P < 0.01 compared to *Apoe*$^{-/-}$; unpaired two-tailed Student's t-test). Data expressed as the ratio of collagen 1 signal to the total vessel area. *Apoe*$^{-/-}$ mice N = 9, *Frs2*^{SMCKO}/*Apoe*$^{-/-}$ mice N = 12. Nuclei were counterstained with DAPI (blue). Scale bar: 62 μm.

Data information: All data shown as mean ± SD (*P < 0.05, **P < 0.01 compared to *Apoe*$^{-/-}$; unpaired two-tailed Student's t-test). A full table of P-values for this figure is shown in Appendix Table S1.

 © 2016 The Authors
