# Supplementary material for: Smooth muscle FGF/TGFβ cross talk regulates atherosclerosis progression
Source: EMBO Mol Med. 2016 May 13;8(7):712–28. doi: 10.15252/emmm.201506181 (PMC4931287; doi:10.15252/emmm.201506181)
Supplement: Supplementary file 3 — Source Data for Expanded View and Appendix [file EMMM-8-712-s006.zip › Source_Data_for_Expanded_View_and_Appendix_figures/201506181_SourceDataFor_EV_Figure2.pdf]

Full unedited gels for EV Figure 2A

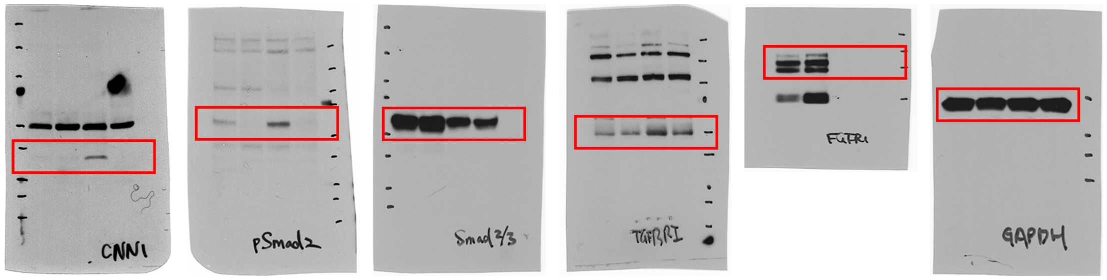

Full unedited gels for EV Figure 2B

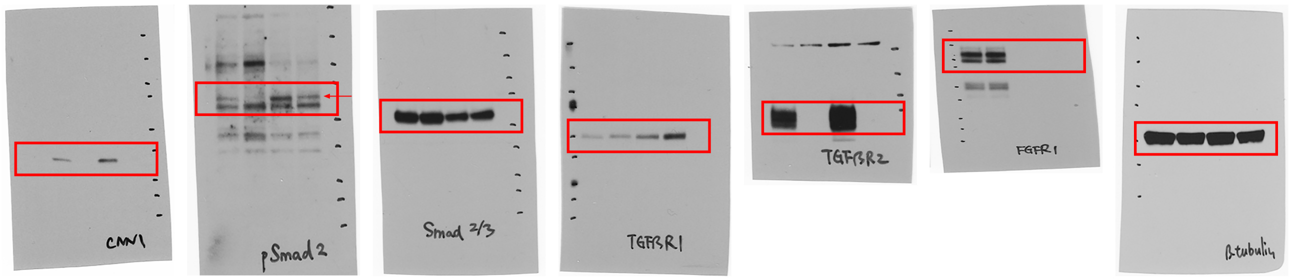

Full unedited gels for EV Figure 2C

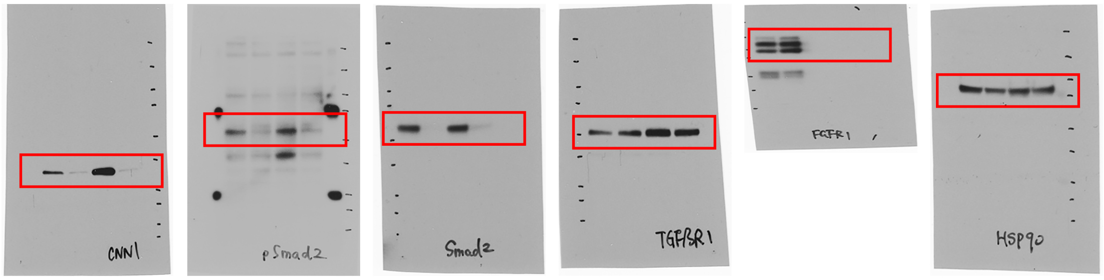

Full unedited gels for EV Figure 2E

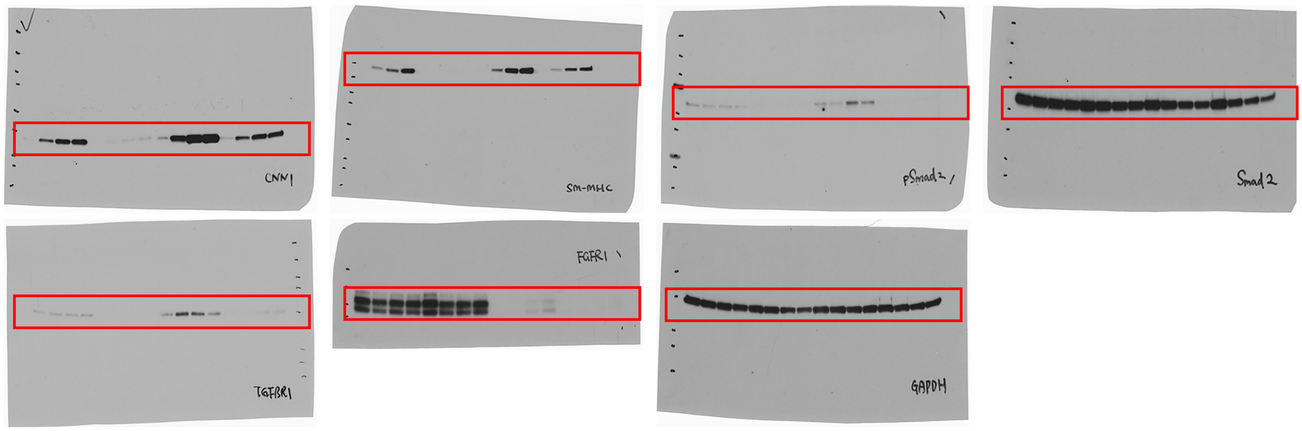

SourceDataForFigureEV2
