# Supplementary figures and images for: Smooth muscle FGF/TGFβ cross talk regulates atherosclerosis progression
Source: EMBO Mol Med. 2016 May 13;8(7):712–28. doi: 10.15252/emmm.201506181 (PMC4931287; doi:10.15252/emmm.201506181)

Full unedited gels for Appendix Figure S1B

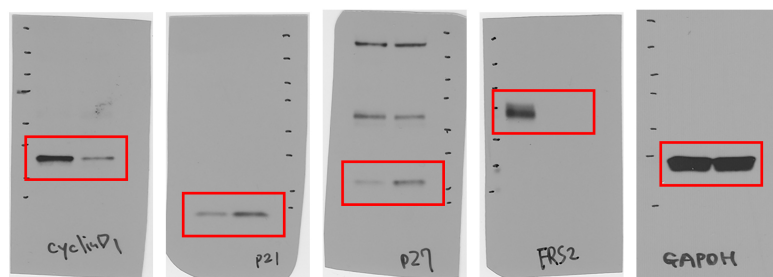

Supplement: Supplementary file 3 — Source Data for Expanded View and Appendix [file EMMM-8-712-s006.zip › Source_Data_for_Expanded_View_and_Appendix_figures/201506181_SourceDataFor_Appendix_FigureS1.pdf]

Full unedited gels for Appendix Figure S2E

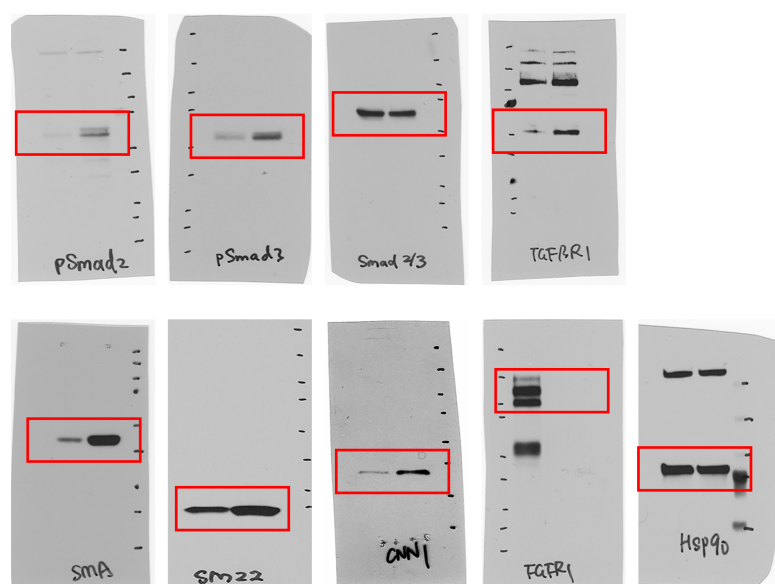

Supplement: Supplementary file 3 — Source Data for Expanded View and Appendix [file EMMM-8-712-s006.zip › Source_Data_for_Expanded_View_and_Appendix_figures/201506181_SourceDataFor_Appendix_FigureS2.pdf]

Full unedited gels for EV Figure 3B

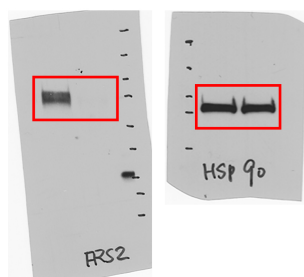

Supplement: Supplementary file 3 — Source Data for Expanded View and Appendix [file EMMM-8-712-s006.zip › Source_Data_for_Expanded_View_and_Appendix_figures/201506181_SourceDataFor_EV_Figure3.pdf]

Full unedited gels for Figure 1C

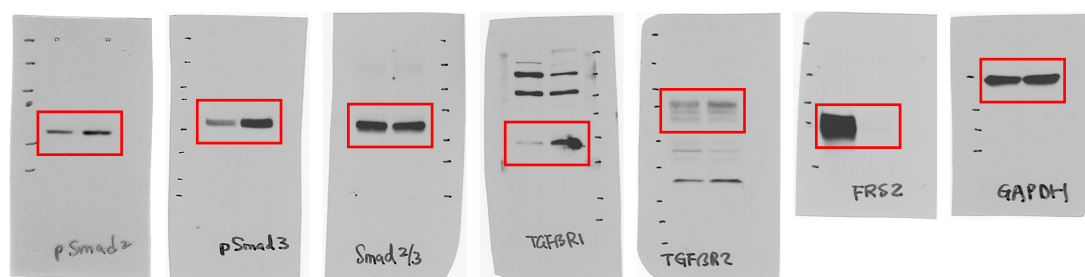

Supplement: Supplementary file 5 — Source Data for Figure 1 [file EMMM-8-712-s003.pdf]

Full unedited gels for Figure 3B

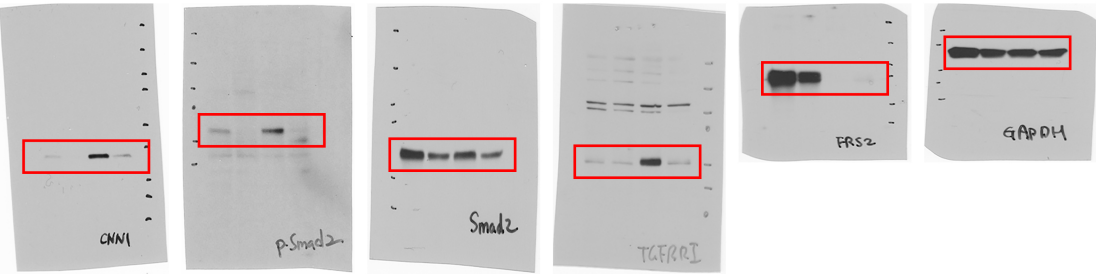

Full unedited gels for Figure 3E

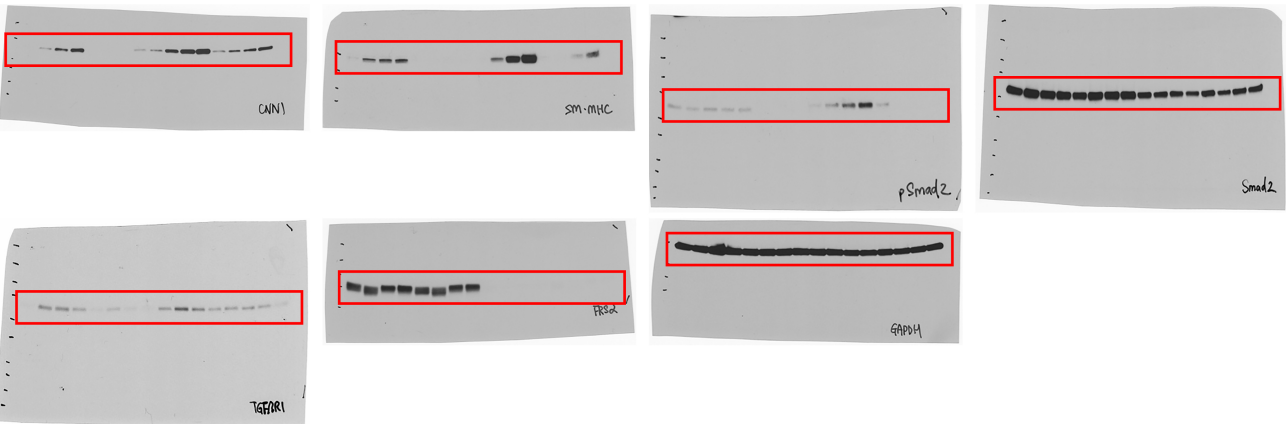

Supplement: Supplementary file 7 — Source Data for Figure 3 [file EMMM-8-712-s005.pdf]
