# Supplementary material for: Smooth muscle FGF/TGFβ cross talk regulates atherosclerosis progression
Source: EMBO Mol Med. 2016 May 13;8(7):712–28. doi: 10.15252/emmm.201506181 (PMC4931287; doi:10.15252/emmm.201506181)
Supplement: Supplementary file 6 — Source Data for Figure 2 [file EMMM-8-712-s004.pdf]

Full unedited gels for Figure 2A

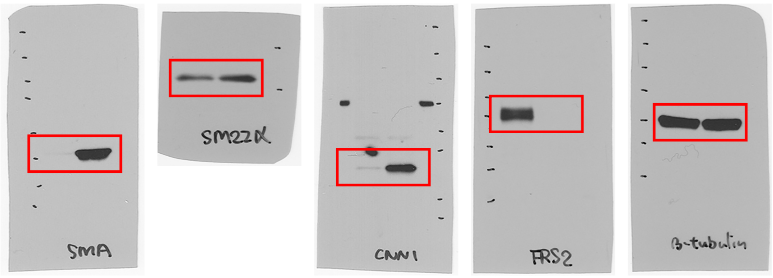

Full unedited gels for Figure 2D

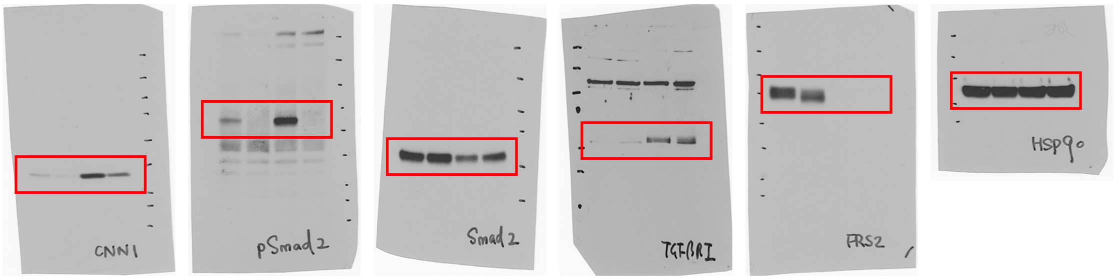

Full unedited gels for Figure 2E

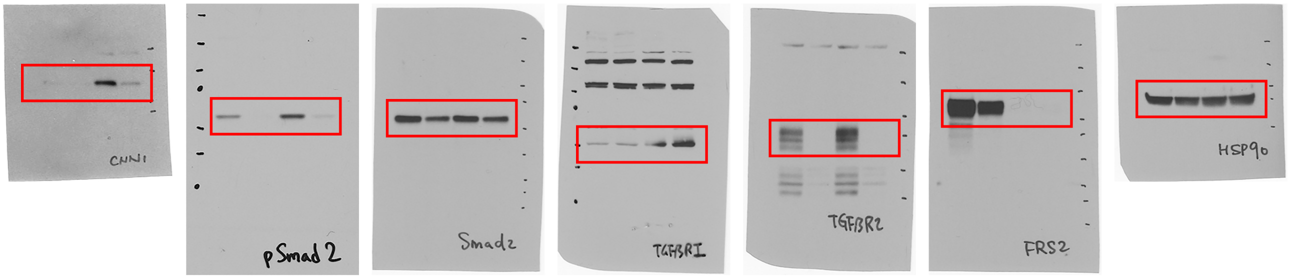

Full unedited gels for Figure 2F

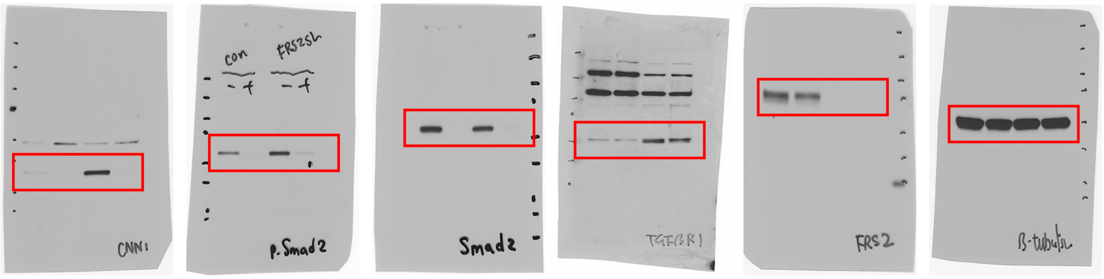

SourceDataForFigure2
